# HPV prevalence and genotype distribution among women in Shandong Province, China: Analysis of 94,489 HPV genotyping results from Shandong’s largest independent pathology laboratory

**DOI:** 10.1371/journal.pone.0210311

**Published:** 2019-01-17

**Authors:** Lingbo Jiang, Xinxin Tian, Dezhi Peng, Liran Zhang, Fengxiang Xie, Chunrui Bi, Rui Wang, Jiajia Wang, Debo Qi

**Affiliations:** 1 Department of Laboratory Medicine, Jinan KingMed Diagnostics, Jinan, Shandong Province, China; 2 Department of Pathology, Jinan KingMed Diagnostics, Jinan, Shandong Province, China; Zhejiang University School of Medicine First Affiliated Hospital, CHINA

## Abstract

**Background:**

Data regarding human papillomavirus (HPV) prevalence and genotype distribution are limited in Shandong Province, China. Therefore, we investigated the recent HPV prevalence and genotype distribution among females in Shandong and aimed to provide comprehensive data to guide HPV-based cervical cancer screening and HPV vaccination for this population of Chinese women.

**Methods:**

HPV testing results of 94,489 females were retrospectively reviewed and extracted from the database of Jinan KingMed Diagnostics, the largest independent pathology laboratory in Shandong Province, China. HPV was detected by a HPV genotyping panel from January 2011 to June 2017. The overall prevalence, age-specific prevalence, and genotype distribution were analyzed.

**Results:**

A total of 26,839 cases (28.4%) were HPV-positive, with 4.3% positive for low- or undetermined-risk HPV (lr-/urHPV)-only, 18.1% positive for high-risk HPV (hrHPV)-only, and 6.1% positive for mixed lr-/urHPV and hrHPV infections. Single HPV infections accounted for 62.8%, while the rest were multiple HPV infections of two or more genotypes. HPV16 (5.8%), HPV52 (5.1%), HPV58 (3.5%), HPV51 (2.6%), and HPV56 (2.3%) were the five most common hrHPV genotypes; while HPV81 (2.8%), HPV53 (2.8%), and HPV6 (2.3%) were the three most common lr-/urHPV genotypes. HPV18 (1.7%) was only the ninth most common hrHPV genotype. HPV16 but not HPV52 was more common in single infections than in multiple infections. The distribution of both mixed lr-/urHPV and hrHPV as well as overall HPV infections demonstrated a bimodal pattern across age groups, of which the first peak appeared in the younger group and the second peak was found in older women. A similar age-specific distribution was observed in multiple infections of three or more subtypes as well. Moreover, the proportion of mixed lr-/urHPV and hrHPV infection significantly increased, while those of lr-/urHPV-only and hrHPV-only infections declined as the number of co-infections increased during the study period.

**Conclusion:**

This large daily clinical practice report shows that HPV prevalence and genotype distribution are different in this population, who had limited cervical cancer screening service, compared to those in developed countries. Therefore, different strategies should be developed for HPV-based cervical cancer screening and vaccine-based HPV prevention in Shandong Province.

## Introduction

Globally, approximately 85% of the estimated 528,000 new cases and about 87% of 266,000 deaths from cervical cancer occurred in developing countries in 2012 [1]. Effective implementations of cervical screening programs have already successfully lowered the incidence and mortality of cervical cancer in developed countries [2–5]. Although there have been several population-based cervical cancer screening programs in China, the majority of the population, especially those in rural areas, do not have access to regular screenings. Therefore, China still has a relatively heavy burden of cervical cancer, and the cervical cancer incidence is significantly underestimated due to the absence of a well-established nationwide cancer registration, especially in rural areas [6, 7].

Human papillomavirus (HPV) infection has been confirmed as the main cause of invasive cervical cancer and its precancerous lesions [8, 9]. More than 200 HPV genotypes have been identified to date, approximately 40 of which infect the genital tract [9]. Approximately 14 genotypes of HPV (HPV16, 18, 31, 33, 35, 39, 45, 51, 52, 56, 58, 59, 66, and 68) are closely associated with cervical, anogenital, and oropharyngeal cancers [9, 10], which, as a result, are classified as high-risk HPV (hrHPV) genotypes [11]. Meanwhile, other common HPV genotypes, such as HPV6, 11, 42, 43, 53, 73, 81, 82, and 83, are indefinitely or not carcinogenic to humans but are causative agents for benign warts in the genital tract, vulva, and perianus; thus, they are classified as low- or undetermined-risk HPV (lr-/urHPV) genotypes [11].

At first, hrHPV detection was an alternative approach to the triage of equivocal abnormal cytology of cervical cancer screening [12, 13]. Currently, several long-term, prospective, controlled trials [14–17] have confirmed that hrHPV testing detects significantly more cervical cancer and its precursors, therefore resulting in lower rates of invasive cancer in subsequent rounds of screening compared with cytology. Thus, hrHPV testing alone has been developed as the primary screening modality in several European countries [18]. Moreover, the improved negative predictive value of HPV testing, which significantly exceeds that of cervical cytology, allows the cervical cancer screening intervals to be safely extended from 3 years to 5 years [18, 19]. Furthermore, HPV vaccination has been demonstrated to provide effective prevention against HPV infection and has been implemented successfully in most developed countries. Two widely used HPV vaccines, Cervarix (HPV16/18) and Gardasil (HPV6/11/16/18), which protect against HPV16 and 18 infection, can prevent more than 70% of cervical cancer occurrence and its precancerous lesions [9]. Additionally, more than 90% of genital warts can be prevented by Gardasil [9]. These two vaccines were approved by the Chinese Food and Drug Administration in July 2016 and June 2017, respectively.

The HPV prevalence and genotype distribution differ between nations [20–23] as well as within a country [16, 24, 25]. Understanding the epidemiological characteristics of HPV infection is of great importance to develop HPV-based cervical cancer screening programs and vaccine-based HPV prevention strategies. Although several studies have been conducted in a few cities of Shandong Province, such as Jinan [24], Qingdao [26], Dongying [27], and Weihai [28, 29], the available data are still insufficient and the HPV-positive rates reported in different studies are inconsistent. Therefore, we conducted a retrospective search of the pathology database of Jinan KingMed Diagnostics (JKD). HPV testing results using a panel of 23 HPV genotypes were documented from January 2011 to June 2017. Overall prevalence, age-specific prevalence, and genotype distribution were calculated and analyzed. This study may provide valuable guidelines for the prevention of cervical cancer and HPV vaccination in the region.

## Materials and methods

### Study population and sample collection

JKD is the largest independent pathology laboratory in Shandong province, China. This study was approved by the institutional review committee of JKD. The review committee waived the need of patients’ consent due to anonymous analyses of the data. The management and publication of patient information in this study was strictly in accordance with the Declaration of Helsinki, including the confidentiality and anonymity. A retrospective database review was conducted to identify all cervical HPV genotyping results reported from January 2011 to June 2017. All the HPV tests were performed at the JKD Genetics Laboratory. The HPV genotyping results and relevant clinical information, including age and clinical history available in the pathology database, were all recorded. Cervical samples for HPV genotyping were collected from more than 500 hospitals, women’s health centers, community clinics, and physical examination centers. These institutes were primarily in suburban and rural areas across 17 cities in Shandong Province, one of the largest and most populous provinces in eastern China. More than 90% of the HPV tests were operated by clinicians during routine cervical screenings, while very few of them were ordered as a triage test after an equivocal abnormal cytology result.

### DNA extraction and HPV genotyping

Exfoliated cervical cells at the orifice of the uterus and endocervical canal were collected using a specialized cervical brush. Then the cervical samples were stored at 4°C in the standard media provided with the panel (Yaneng Bioscience, Shenzhen, China) and shipped to the JKD Genetics Laboratory for HPV testing within 48 h. DNA isolation and purification were conducted according to the manufacturer’s instructions (Yaneng Bioscience, Shenzhen, China).

All HPV tests were performed with a HPV genotyping panel (polymerase chain reaction (PCR)-reverse dot blot hybridization method; Yaneng Bioscience, Shenzhen, China). This multiplex PCR technique can detect 14 hrHPV genotypes (16, 18, 31, 33, 35, 39, 45, 51, 52, 56, 58, 59, 66, and 68) and 9 lr-/urHPV genotypes (6, 11, 42, 43, 53, 73, 81, 82, and 83). As reported previously [30–32], HPV DNA was amplified by the mixture of GP5+/GP6+ consensus and type-specific primers after extraction from the specimens. The PCR cycling conditions were as follows: initial denaturation at 95°C for 5 min, then 40 cycles at 94°C for 30 s, 42°C for 90 s, 72°C for 30 s, and a final extension at 72°C for 5 min [31, 32]. The HPV type-specific probes were immobilized on nylon membranes, which were used for reverse-blot hybridization and detection of all HPV genotypes in a single assay. The hybridization was performed and visualized by assessment protocols, according to the manufacturer’s instructions. The quality and integrity of extracted DNA was monitored by β-actin gene detection in each replicate tube. Sterile water was used as the negative control, and specimens with known HPV genotypes were used as the positive control.

### Statistical analysis

Data were analyzed with SAS 9.4 (SAS Institute, Cary, NC). All subjects were divided into four age groups (≤30, 31–50, >50 years, and unknown ages) to calculate the overall HPV genotype prevalence, or into seven age groups (≤20, 21–30, 31–40, 41–50, 51–60, >60 years, and unknown ages) for the other analyses. The age (women with unknown ages were excluded) and co-infection number trends of HPV infection were analyzed by the Cochran–Armitage trend test. *P* < 0.05 was considered to be statistically significant.

## Results

### Overall and age-specific HPV prevalence

From January 2011 to June 2017, a total of 94,640 samples were collected for HPV infection detection at the JKD Genetics Laboratory, but only 94,489 cases (99.8%) were suitable for final statistical analysis due to the presence of β-actin. The HPV test results demonstrated that 26,839 cases were HPV-positive, and the overall prevalence of detectable HPV infection was 28.4%, with 4.3% positive for lr-/urHPV-only, 18.1% positive for hrHPV-only, and 6.1% positive for mixed lr-/urHPV and hrHPV infections.

After dividing the subjects into seven age groups (≤20, 21–30, 31–40, 41–50, 51–60, >60 years, and unknown ages), the HPV infection prevalence in each subgroup was calculated. As shown in Table 1, a different age-specific prevalence was observed in lr-/urHPV-only infection (*Z*trend = -14.00, *P*trend < 0.001). The highest prevalence was found in the youngest age group (9.8%), and the infection rate declined gradually as the age increased (21–30, 31–40, 41–50, 51–60, and >60 years). In contrast, the distribution of mixed lr-/urHPV and hrHPV infection showed a bimodal pattern in different age groups (Ztrend = -27.50, *P*trend < 0.001). The highest infection rate was found in women ≤20 years old (27.7%), which declined gradually and reached its lowest point in the group of 41–50 year olds (4.0%), before another increasing trend in the older age groups (5.6% for the group of 51–60 year olds and 5.9% for women >60 years old). A similar age-specific prevalence pattern was found in overall HPV infection (Ztrend = -27.78, *P*trend < 0.001), with the first peak in women ≤20 years old (56.0%), and the second peak in women in their fifties (25.9%). Besides, hrHPV-only infection was very common, with the infection rate exceeding 15% in all age groups (Ztrend = -8.10, *P*trend < 0.001).

**Table 1:** Age-stratified HPV prevalence in 94,489 tested women.

Age Group, y	Total No.	lr/urHPV-only		hrHPV-only		Mixed lr-/urHPV and hrHPV		Total	
		Positive No.	Positive, % (95% CI)	Positive No.	Positive, % (95% CI)	Positive No.	Positive, % (95% CI)	Positive No.	Positive, % (95% CI)
≤20	1,633	160	9.8 (8.4–11.2)	302	18.5 (16.6–20.4)	453	27.7 (25.6–29.9)	915	56.0 (53.6–58.4)
21–30	18,469	1059	5.7 (5.4–6.1)	3631	19.7 (19.1–20.2)	1801	9.8 (9.3–10.2)	6,491	35.1 (34.5–35.8)
31–40	27,616	1084	3.9 (3.7–4.2)	4923	17.8 (17.4–18.3)	1286	4.7 (4.4–4.9)	7,293	26.4 (25.9–26.9)
41–50	29,230	1034	3.5 (3.3–3.7)	4944	16.9 (16.5–17.3)	1179	4.0 (3.8–4.3)	7,157	24.5 (24.0–25.0)
51–60	9,509	346	3.6 (3.3–4.0)	1586	16.7 (15.9–17.4)	532	5.6 (5.1–6.1)	2,464	25.9 (25.0–26.8)
>60	2,381	59	2.5 (1.9–3.1)	368	15.5 (14.0–16.9)	140	5.9 (4.9–6.8)	567	23.8 (22.1–25.5)
Unknown	5,651	282	5.0 (4.4–5.6)	1305	23.1 (22.0–24.2)	365	6.5 (5.8–7.1)	1,952	34.5 (33.3–35.8)
Total	94,489	4024	4.3 (4.1–4.4)	17059	18.1 (17.8–18.3)	5756	6.1 (5.9–6.2)	26,839	28.4 (28.1–28.7)

### Overall prevalence of all HPV genotypes

The prevalence of each HPV genotype is shown in Table 2. The most common hrHPV genotype detected was HPV16 (5.8%), followed by HPV52 (5.1%), HPV58 (3.5%), HPV51 (2.6%), and HPV56 (2.3%). HPV18 was only the ninth most common hrHPV genotype detected. Additionally, in the subgroup analysis by age (≤30, 31–50, and >50 years old), HPV16, 52, and 58 were consistently the three most common hrHPV genotypes in all age groups. Slight variations were observed in the prevalence of HPV51, 56, 59, 66, 18, 33, 39, and 31. HPV35 and 45 were always the two least frequent hrHPV genotypes in all age groups.

**Table 2:** The prevalence of all HPV genotypes in 94,489 tested women.

	Genotype	≤30 y (n = 20,102)		31–50 y (n = 56,846)		>50 y (n = 11,890)		Unknown (n = 5,651)		All Ages (n = 94,489)	
		Positive No. (%)	95% CI	Positive No. (%)	95% CI	Positive No. (%)	95% CI	Positive No. (%)	95% CI	Positive No. (%)	95% CI
hrHPV genotype	16	1,532 (7.6)	7.3–8.0	2,896 (5.1)	4.9–5.3	595 (5.0)	4.6–5.4	425 (7.5)	6.8–8.2	5,448 (5.8)	5.6–5.9
	52	1,415 (7.0)	6.7–7.4	2,510 (4.4)	4.2–4.6	557 (4.7)	4.3–5.1	373 (6.6)	6.0–7.2	4,855 (5.1)	5.0–5.3
	58	1,009 (5.0)	4.7–5.3	1,643 (2.9)	2.8–3.0	398 (3.3)	3.0–3.7	222 (3.9)	3.4–4.4	3,272 (3.5)	3.3–3.6
	51	847 (4.2)	3.9–4.5	1,149 (2.0)	1.9–2.1	271 (2.3)	2.0–2.5	183 (3.2)	2.8–3.7	2,450 (2.6)	2.5–2.7
	56	637 (3.2)	2.9–3.4	1,065 (1.9)	1.8–2.0	292 (2.5)	2.2–2.9	161 (2.8)	2.4–3.3	2,155 (2.3)	2.2–2.4
	68	612 (3.0)	2.8–3.3	1,017 (1.8)	1.7–1.9	216 (1.8)	1.6–2.1	152 (2.7)	2.3–3.1	1,997 (2.1)	2.0–2.2
	59	577 (2.9)	2.6–3.1	692 (1.2)	1.1–1.3	145 (1.2)	1.0–1.4	91 (1.6)	1.3–1.9	1,505 (1.6)	1.5–1.7
	66	568 (2.8)	2.6–3.1	843 (1.5)	1.4–1.6	204 (1.7)	1.5–1.9	132 (2.3)	1.9–2.7	1,747 (1.8)	1.8–1.9
	18	541 (2.7)	2.5–2.9	823 (1.4)	1.3–1.5	164 (1.4)	1.2–1.6	120 (2.1)	1.7–2.5	1,648 (1.7)	1.7–1.8
	33	443 (2.2)	2.0–2.4	683 (1.2)	1.1–1.3	158 (1.3)	1.1–1.5	102 (1.8)	1.5–2.2	1,386 (1.5)	1.4–1.5
	39	393 (2.0)	1.8–2.1	665 (1.2)	1.1–1.3	139 (1.2)	1.0–1.4	92 (1.6)	1.3–2.0	1,289 (1.4)	1.3–1.4
	31	378 (1.9)	1.7–2.1	705 (1.2)	1.1–1.3	154 (1.3)	1.1–1.5	96 (1.7)	1.4–2.0	1,333 (1.4)	1.3–1.5
	35	247 (1.2)	1.1–1.4	540 (0.9)	0.9–1.0	136 (1.1)	1.0–1.3	74 (1.3)	1.0–1.6	997 (1.1)	1.0–1.1
	45	173 (0.9)	0.7–1.0	224 (0.4)	0.3–0.4	44 (0.4)	0.3–0.5	37 (0.7)	0.4–0.9	478 (0.5)	0.5–0.6
Lr-/urHPV genotype	6	1,161 (5.8)	5.5–6.1	752 (1.3)	1.2–1.4	125 (1.1)	0.9–1.2	112 (2.0)	1.6–2.3	2,150 (2.3)	2.2–2.4
	11	809 (4.0)	3.8–4.3	511 (0.9)	0.8–1.0	96 (0.8)	0.6–1.0	82 (1.5)	1.1–1.8	1,498 (1.6)	1.5–1.7
	81	737 (3.7)	3.4–3.9	1,369 (2.4)	2.3–2.5	372 (3.1)	2.8–3.4	185 (3.3)	2.8–3.7	2,663 (2.8)	2.7–2.9
	53	712 (3.5)	3.3–3.8	1,313 (2.3)	2.2–2.4	385 (3.2)	2.9–3.6	200 (3.5)	3.1–4.0	2,610 (2.8)	2.7–2.9
	43	601 (3.0)	2.8–3.2	855 (1.5)	1.4–1.6	182 (1.5)	1.3–1.8	100 (1.8)	1.4–2.1	1,738 (1.8)	1.8–1.9
	42	586 (2.9)	2.7–3.1	834 (1.5)	1.4–1.6	238 (2.0)	1.7–2.3	120 (2.1)	1.7–2.5	1,778 (1.9)	1.8–2.0
	73	102 (0.5)	0.4–0.6	116 (0.2)	0.2–0.2	30 (0.3)	0.2–0.3	18 (0.3)	0.2–0.5	266 (0.3)	0.2–0.3
	82	67 (0.3)	0.3–0.4	100 (0.2)	0.1–0.2	8 (0.1)	0.1–0.1	12 (0.2)	0.1–0.3	187 (0.2)	0.2–0.2
	83	65 (0.3)	0.2–0.4	140 (0.2)	0.2–0.3	55 (0.5)	0.3–0.6	8 (0.1)	0.0–0.2	268 (0.3)	0.2–0.3

For the lr-/urHPV genotypes, HPV81 (2.8%), HPV53 (2.8%), and HPV6 (2.3%) were the three most common types (Table 2). Obvious differences among the different age groups were found for the lr-/urHPV genotype distribution. For women younger than 30 years old, HPV6 (5.8%) and HPV11 (4.0%) were the two most common lr-/urHPV genotypes. Whereas, for women older than 30 years old (31–50 and >50 years old), the two most common genotypes were HPV81 and 53. HPV73, 82, and 83 were the three least frequently detected lr-/urHPV genotypes in all age groups.

### Age-specific distribution of single and multiple HPV infections

The age-specific distribution of single and multiple (two or more) HPV infections is summarized in Table 3. Single HPV infections accounted for 62.8% of the 26,839 HPV-positive cases. For women infected with multiple HPV genotypes, 6,017 (22.4%), 2,358 (8.8%), 885 (3.3%), and 724 (2.7%) cases had double, triple, quadruple, and ≥ five HPV genotype infections, respectively. Significant differences were observed in the age-specific distribution of single genotype infections (Ztrend = 16.96, *P*trend < 0.001). Starting from 33.7% in the youngest group, the infection rate increased gradually and reached its peak in the 41–50 year olds, and then it remained at a relatively high level for women >50 years old. However, an inverse distribution was observed in triple (Ztrend = -8.91, *P*trend < 0.001), quadruple (Ztrend = -9.65, *P*trend < 0.001), and ≥ five (Ztrend = -13.74, *P*trend < 0.001) HPV genotype infections. The highest infection rate was found in women ≤20 years old (17.6%, 9.6%, and 14.4%, respectively), which declined gradually as the age increased, reached the lowest levels in the group of 41–50 year olds (6.6%, 2.2%, and 1.1%, respectively), and then rose again in women >50 years old. Besides, no obvious difference was observed in the distribution of double genotype infections, with proportions exceeding 20% in all age groups (Ztrend = -4.10, *P*trend < 0.001).

**Table 3:** Age-specific distribution of single and multiple HPV infections.

Age Group, y	Single HPV genotype		Double HPV genotypes		Triple HPV genotypes		Quadruple HPV genotypes		≥Five HPV genotypes		Total No.
	No.	Proportion (%)	No.	Proportion (%)	No.	Proportion (%)	No.	Proportion (%)	No.	Proportion (%)	
≤20	308	33.7	226	24.7	161	17.6	88	9.6	132	14.4	915
21–30	3,598	55.4	1,567	24.1	737	11.4	315	4.9	274	4.2	6,491
31–40	4,837	66.3	1,624	22.3	555	7.6	178	2.4	99	1.4	7,293
41–50	4,942	69.1	1,506	21.0	475	6.6	158	2.2	76	1.1	7,157
51–60	1,585	64.3	545	22.1	212	8.6	63	2.6	59	2.4	2,464
>60	324	57.1	116	20.5	66	11.6	28	4.9	33	5.8	567
Unknown	1,261	64.6	433	22.2	152	7.8	55	2.8	51	2.6	1,952
Total	16,855	62.8	6,017	22.4	2,358	8.8	885	3.3	724	2.7	26,839

### Genotype distribution in single and multiple HPV infections

**Fig 1:**
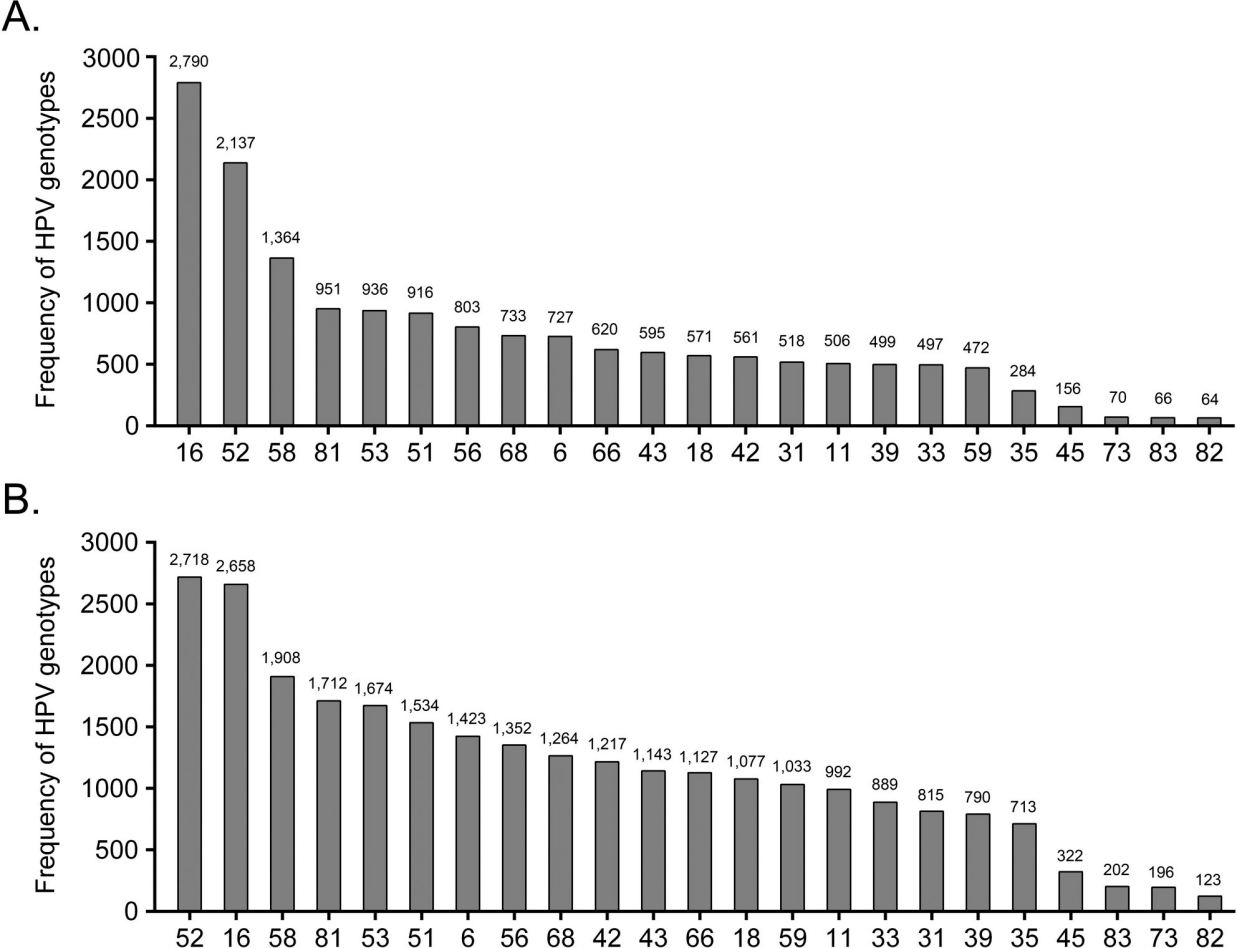
**Distribution of HPV genotypes in single (A) and multiple (B) HPV infections**.

The genotype distribution for single and multiple HPV infections is presented in Fig 1. For individuals infected with a single HPV genotype, the most commonly detected hrHPV genotype was HPV16 (16.6%), followed by HPV52 (12.7%), HPV58 (8.1%), HPV51 (5.4%), and HPV56 (4.8%). In contrast, HPV52 (10.1%) was the most commonly detected hrHPV genotype for multiple HPV infection, followed by HPV16 (9.9%), HPV58 (7.1%), HPV51 (5.7%), and HPV56 (5.0%). In single and multiple HPV-infected individuals, HPV35 and 45 were consistently the two least frequent hrHPV genotypes. As for the lr-/urHPV genotypes, HPV81 and 53 were the two most common genotypes, while HPV73, 83, and 82 were the three least frequent types both in the single and multiple HPV infections.

### Distribution of lr-/urHPV-only, hrHPV-only, and mixed lr-/urHPV and hrHPV infections in co-infections with different numbers

As shown in Table 4, different distributions of lr-/urHPV-only (Ztrend = -33.61, *P*trend < 0.001), hrHPV-only (Ztrend = -72.30, *P*trend < 0.001), and mixed lr-/urHPV and hrHPV infections (Ztrend = 33.49, *P*trend < 0.001) were observed in co-infections with different numbers. The proportion of lr-/urHPV-only and hrHPV-only infections had the highest level in single HPV infection (21.0% and 79.0%, respectively), and it declined gradually as the number of co-infections increased. In contrast, the proportion of mixed lr-/urHPV and hrHPV infection was the lowest in double HPV genotype infections (45.0%), but it rose dramatically as the number of co-infections increased.

**Table 4:** Distribution of lr-/urHPV-only, hrHPV-only, and mixed lr-/urHPV and hrHPV infections in co-infections with different numbers.

Number of co-infections	lr-/urHPV-only		hrHPV-only		Mixed lr-/urHPV and hrHPV		Total No.
	No.	Proportion (%)	No.	Proportion (%)	No.	Proportion (%)	
Single HPV genotype	3,537	21.0	13,318	79.0	–	–	16,855
Double HPV genotypes	442	7.3	2,867	47.6	2,708	45.0	6,017
Triple HPV genotypes	38	1.6	687	29.1	1,633	69.3	2,358
Quadruple HPV genotypes	6	0.7	153	17.3	726	82.0	885
≥Five HPV genotypes	1	0.1	34	4.7	689	95.2	724
Total	4,024	15.0	17,059	63.6	5,756	21.4	26,839

## Discussion

This study provided an enormous amount of HPV genotyping data from a largely unscreened Chinese female cohort in Shandong Province, China. The results revealed that the overall HPV-positive rate was 28.4%, with 4.3% positive for lr-/urHPV-only, 18.1% positive for hrHPV-only, and 6.1% positive for mixed lr-/urHPV and hrHPV infections, which were consistent with the results from several large-scale investigations of HPV prevalence and genotype distribution among other Chinese populations [16, 24, 33, 34]. The results showed that China had a higher level of HPV infection prevalence than other countries where cervical cancer screening was well established [35, 36]. The prevalence of hrHPV has been shown to be closely related to the risk as well as incidence of cervical cancer [37]. Thus, the high hrHPV-positive rate in our study indicated the inadequacy of routine cervical screening, which reflects the high cervical cancer incidence in Shandong Province, China.

Previous studies have demonstrated that HPV prevalence varies considerably in women from different regions and nations [16, 20–24, 33, 34]. A recent study, which investigated the HPV prevalence among more than 1 million women without cervical abnormalities, showed that Western Asia (1.7%) and North America (4.7%) have the lowest rates of HPV infection, while East Africa (33.6%) and the Caribbean (35.4%) have the highest rates of HPV infection [20]. HPV prevalence also varies in China because of its large population and territory. The hrHPV prevalence differs from 9.9% to 31.9% in various areas of China [16, 24, 25, 33, 34]. Moreover, several recent studies have shown that the HPV prevalence also varies significantly within Shandong Province. For instance, HPV-positive rates range from 6.9% to 32.2% in Jinan [24], Qingdao [26], Dongying[27], and Weihai [28, 29].

The age-stratified analyses indicated a bimodal pattern of hrHPV prevalence among different age groups. The first peak was in the younger age group, while the second peak was in older women from China [24, 25, 33, 34] and other populations [21, 22]. In this study, both the mixed lr-/urHPV and hrHPV infection as well as overall HPV prevalence reached a peak in women ≤20 years old, gradually declined in the middle age groups, and then increased slightly in women >50 years old. Similarly, this bimodal pattern was also observed in multiple infections of three or more genotypes. The age-specific distributions in this study and extant studies [22, 24, 33, 34] reflect the natural history of HPV infection. Young women, who are sexually active just after beginning sexual relations and whose immune systems have not been sensitized, are sensitive to HPV infections. Therefore, the infection rate is much higher. Also, an increased number of younger people are infected by several genotypes; thus, multiple and mixed lr-/urHPV and hrHPV infections are more frequent in younger groups than in older women. Nevertheless, most HPV infections in young women are temporary, and are cleared or suppressed within one or two years in 70–91% of the cases [8, 38]. Thus, the positive rate of mixed lr-/urHPV and hrHPV infection as well as the proportion of multiple infections gradually declines as age increases. If the immune system fails to clear the virus, a few persistent infections of hrHPV may lead to the occurrence of cervical cancer [8–10]. The slight increase of mixed lr-/urHPV and hrHPV infection as well as overall infection rates in older women and the increased proportion of older women with multiple infections of three or more genotypes might reflect an impaired immune response as a result of hormonal changes at menopause. In this case, latent HPV infections might be reactivated, and new HPV infections might occur [22, 39]. Another possible explanation for the second peak might be attributed to changes in the sexual behavior of women and their partners in middle age [22].

It is necessary to know the HPV genotype distribution to select the spectrum of HPV genotypes for both HPV-based screening and vaccine development. In this study, the HPV genotyping panel of Yaneng Bioscience, a clinical *in vitro* diagnostic tool approved by the Chinese Food and Drug Administration in 2008 (approval No. 2008–340099), was employed. Our recent study [30] showed that this genotyping method correlates well with Hybrid Capture II (HCII) testing. The hrHPV-positive rate was 94.8% for HCII testing, and a 93.9% positive rate was found for this HPV genotyping panel in invasive cervical cancer.

The HPV genotype distribution varies geographically in the general population, in cervical cancer patients, as well as in patients with precancerous lesions. Our study found that HPV16 (5.8%), HPV52 (5.1%), HPV58 (3.5%), HPV51 (2.6%), and HPV56 (2.3%) were the five dominant hrHPV genotypes in the population. This genotype distribution pattern is similar to previously reported data from several other Chinese population-specific investigations [24, 26–29, 33]. These studies reported that both HPV52 and 58 are more common among the general population in China [24, 26–29, 33] than in developed countries [22, 40]. Moreover, several studies [26, 32, 41–43] have indicated that HPV58 and 52 are also more common in patients with cervical cancer or its precursors in China than in other countries [32, 40, 44, 45]. A recent meta-analysis has demonstrated that the attributions of HPV52 and 58 to cervical intraepithelial neoplasia (CIN) and invasive cancer in Eastern Asia were 2.5–2.8- and 3.7–4.9-fold higher than elsewhere [46]. Zeng *et al*. [33] also have found that HPV52 instead of HPV16 was more common in younger women (<30 years old or 30–49 years old) than in women ≥50 years old in China. However, no obvious differences of the most widespread hrHPV genotypes were observed among the women included in this study, as HPV16, 52, and 58 were consistently the three most common hrHPV genotypes in all age groups.

Apart from HPV16, HPV18 is known as the second most common oncogenic genotype in the general female population [20, 22] and in patients with cervical cancer or its precursors worldwide [44, 45, 47]. A triage test identifying CIN3 or worse in HPV-positive women has reported that the detection of HPV16/18 is equivalent to the detection of atypical squamous cells of undetermined significance or worse alone in terms of sensitivity (59.5% vs. 52.8%) and positive predictive value (15.5% vs. 14.1%) [35]. A cervical cancer prevention program based only on cytology is not always viable in regions lacking resources in China, due to the scarcity of qualified cytopathologists and standard systems for cytology quality control. The hrHPV testing is a feasible alternative to cytology for primary screening because of its potential of high-throughput automation and reduced dependence on qualified cytopathologists. Nonetheless, HPV18 was only the ninth most common hrHPV genotype detected in our study, which was consistent with several previous studies in China [24, 26, 27, 29, 33]. The proportion of invasive cervical cancer cases associated with HPV16 and/or 18 was only 68% in Eastern Asia, including China [47]. Zhang *et al*. [32] have reported that only 49.1% of cervical squamous cell carcinoma and 65.1% of adenocarcinoma were attributed to HPV16/18 in Qindao, a city in eastern Shandong. When HPV16/18 is used for the triage of hrHPV-positive results, the efficacy of hrHPV testing as the primary screening modality might be limited in China. Further studies are warranted to determine whether additional predominant hrHPV genotypes should be included in triage for hrHPV-positive women in this region.

Furthermore, this study showed that the three most common lr-/urHPV genotypes were HPV81 (2.8%), HPV53 (2.8%), and HPV6 (2.3%), inconsistent with previous results showing that HPV6 was the most frequently detected lr-/urHPV genotype among Americans (2.9%), followed by HPV11 and 61 [21]. Both Cervarix and Gardasil vaccines protect against hrHPV infection by HPV16 and 18, and Gardasil additionally defends against lr-/urHPV infection by HPV6 and 11. However, these two vaccines provide no protection against some of the other hrHPV genotypes found in at least 25% of cervical cancers or 10% of genital warts [48]. The findings in this study indicated that in addition to HPV16 and 18, the genotypic spectrum of the next-generation HPV vaccine for women in Shandong Province should include other common hrHPV genotypes, such as HPV52 and 58, and lr-/urHPV genotypes, like HPV81 and 53. The nine-valent HPV vaccine (HPV6/11/16/18/31/33/45/52/58), which can prevent the occurrence of more than 90% of cervical cancer and its precursors [49], should be approved as soon as possible in China to prevent HPV infections by predominant genotypes effectively [50].

Investigation of the prevalence of multiple HPV infection is also of great significance to understand its carcinogenic role in cervical cancer. Although women co-infected with HPV16 and other hrHPV genotypes have no higher risk for cervical cancer than those infected with HPV16 alone [48], several studies have shown that HPV infection with multiple genotypes increases the duration of infection as well as the risk of cervical cancer and cervical precancerous lesions [51, 52]. In our study, multiple HPV infection accounted for 37.2% of all HPV-positive cases. This rate was comparable to rates reported in Weihai (36.9%) [29] and Qingdao (37.4%) [26], but much higher than those reported in other Chinese [24, 33, 42] and international [21] populations. This study also found that HPV52 (10.1%) was the most commonly detected hrHPV genotype in multiple rather than single HPV infection. Moreover, the proportions of lr-/urHPV-only and hrHPV-only infections gradually declined, while mixed lr-/urHPV and hrHPV infection showed an inverse distribution pattern as the number of co-infections increased. The potential competitive and/or cooperative oncogenic effects of multiple HPV genotype infection need to be further investigated to help develop HPV prophylactic vaccines.

Although this study provided large-scale information on the recent HPV prevalence and genotype distribution in Shandong Province, China, several limitations existed. First, most cervical screening tests received by JKD were either for HPV testing alone or cytology alone, while HPV and cytology co-testing were rare. Cervical cytology or histology results were not available for women included in the study; therefore, we were unable to correlate HPV infection and genotype distribution to different cervical abnormalities. It still remains as a clinical dilemma to manage appropriately such a large cohort of HPV-positive women without cytological evidence of cervical cancer or precancerous lesions. Second, detailed information on geographic, cultural, economic, and other backgrounds of this population was diverse; however, it was not documented in this study. Therefore, we were unable to specify the effect of these different backgrounds on the prevalence of HPV infection. Third, very few cases had HPV testing due to an equivocal abnormal cytology result. This might lead to overestimation of the actual prevalence of HPV infection, which also has been shown in several other Chinese studies [24, 33, 34].

## Conclusion

To the best of our knowledge, this study was the most comprehensive routine clinical assessment to reveal the prevalence of HPV infection and genotype distribution in a female cohort in Shandong Province, China. This study confirmed a high HPV prevalence and indicated a high cervical cancer incidence in this region, where well-organized screening has not been well established and prophylactic vaccines against HPV have not been available. The results showed that HPV16, 52, and 58 were the three most common hrHPV genotypes. These findings were consistent with those among other Chinese populations. However, the HPV genotype distribution pattern was clearly distinct from those found in developed countries. Further trials are needed to determine whether a different triage strategy should be developed when hrHPV testing is adopted as the primary screening modality in China. Meanwhile, the genotypic spectrum of the next-generation HPV vaccine for Chinese women should include other predominant hrHPV genotypes, such as HPV52 and 58.
